# Prevalence of autoimmune thyroiditis among children with autoimmune hepatitis

**DOI:** 10.1186/s13052-024-01639-4

**Published:** 2024-04-18

**Authors:** Engy Adel Mogahed, Hend M. Soliman, Dalia Saber Morgan, Hoda Mohammed Abd Elaal, Rasha Abd El Razek Mahmoud Khattab, Ragaey A. Eid, Mahmoud Hodeib

**Affiliations:** 1 Department of Pediatrics, New Children Hospital, Cairo University, Cairo, Egypt; 2 Department of Pediatrics, Faculty of Medicine, Beni-Suef University, Beni Suef, Egypt; 3 Immunology- Clinical Pathology Department, Faculty of Medicine, Beni-Suef University, Beni Suef, Egypt; 4 Department of Gastroenterology, Hepatology and Infectious Diseases ( Tropical Medicine department), Faculty of Medicine, Beni-Suef University, Beni Suef, Egypt

**Keywords:** Autoimmune hepatitis, Children, Autoimmune diseases, Autoimmune thyroiditis

## Abstract

**Background:**

Autoimmune hepatitis (AIH) is an organ specific autoimmune disease, which can manifest at any age of life. there is a high prevalence of extrahepatic autoimmune diseases in patients with AIH. Autoimmune thyroid diseases (ATDs) are the most frequent extrahepatic autoimmune disorders among patients with AIH. Aim of work is to detect the frequency of ATDs among Egyptian children with AIH.

**Methods:**

This research is a cross-sectional study conducted on 58 children with AIH aged ≤ 18 years. All patients were tested for free triiodothyronine (FT3), free tetraiodothyronine (FT4), thyroid stimulating hormone (TSH), anti-thyroid peroxidase (anti-TPO) and antithyroglobulin (anti-TG). Thyroid ultrasound (US) and thyroid scan were performed for patients with abnormal thyroid profile, borderline values, positive anti-TPO or anti-TG.

**Results:**

The mean ± standard deviation (SD) for the age of the patients was 11.3 ± 4.5 years. Out of 58 patients of AIH, 28 patients (48.3%) had associated other autoimmune diseases. Autoimmune thyroiditis was the most common associated autoimmune disease being present in 10 patients (17.2%). The thyroid status of AIT patients showed that 6 patients (60%) were euthyroid, 3 patients (30%) had subclinical hypothyroidism and only one patient (10%) was hyperthyroid.

**Conclusion:**

Autoimmune hepatitis in Egyptian children is commonly associated with other autoimmune diseases. Autoimmune thyroiditis is the most common to be associated with AIH in pediatric patients. As it is not usually clinically manifesting, regular screening for AIT in children with AIH is mandatory.

## Introduction

Autoimmune hepatitis (AIH) is an organ-specific autoimmune disease of unknown etiology that manifests as a chronic inflammatory disease of the liver, typically characterized by periportal inflammation, elevated autoantibodies, and hypergammaglobulinemia [1]. A variety of clinical presentations can be observed, ranging from mild, almost subclinical disease to fulminant hepatitis [2, 3]. While the pathogenesis is not fully understood, the current hypothesis is that an environmental agent is thought to trigger a dysregulated T-cell response against autoantigens in genetically susceptible individuals [4].

There is a high prevalence of concurrent extrahepatic autoimmune diseases in children with AIH (42%) [5]. It showed a significant association with autoimmune thyroid disease, followed by autoimmune skin disease, celiac disease, and vasculitis. All patients with an extrahepatic autoimmune disease should be assessed for the concomitant presence of an asymptomatic AIH [6]. Autoimmune thyroid diseases, including Hashimoto’s thyroiditis (HT), are common organ-specific autoimmune diseases that frequently coexist with other autoimmune disorders [7]. They are the most frequent extrahepatic autoimmune diseases among patients with AIH (16.01%) [8, 9].

Autoimmune thyroid diseases are the most common thyroid disorders in pediatrics and adolescence [10]. Patients may have euthyroid, or they may have subclinical or overt hypothyroidism depending on the severity of the immunologic damage [11]. The most common age at presentation is adolescence, but the disease may occur at any time, rarely even in children under one year [12].

Children and adolescents are primarily asymptomatic; the majority are female, pubertal, and euthyroid. Diagnosing at an early stage offers the opportunity for a timely intervention [13].

Measurement of the serum thyroid stimulating hormone (TSH) concentration is the best initial screening test for the presence of primary hypothyroidism. If the TSH is elevated, then evaluation of the serum-free tetraiodothyronine (FT4) concentration will distinguish whether the child has subclinical (average FT4) or overt (low FT4) hypothyroidism [13]. A diagnosis of autoimmune thyroiditis (AIT) is made by the demonstration of an elevated concentration of antithyroglobulin antibodies (anti-TG Abs) and/or antithyroid peroxidase antibodies (anti-TPO Abs) in serum [11, 14].

The aim of this study is to screen children with AIH to detect the frequency of associated AIT. Our secondary objective was to correlate AIT with AIH activity.

## Patients and methods

This cross-sectional study was conducted at two Pediatric Hepatology centers (Beni-Suef University Hospital & Cairo University Hospital, Egypt). The study included all children with established diagnoses of AIH ≤ 18 years of both sexes, either they were newly presenting cases or old cases coming for follow-up from October 2020 to October 2022. This research was approved by the Research Ethics Committee Review Board of the Faculty of Medicine, Beni-Suef University [Approval NO: FMBSUREC/30042019}. Informed consent was obtained from all the study patients or their guardians before data collection. Confidentiality in handling the database was guaranteed. The privacy of participants was ensured. *To screen for associated AIT, FT3, FT4, TSH, anti-TPO, and anti-TG were done for all patients at the time of study enrollment, regardless of whether they had a new AIH diagnosis or established disease.*

Patients with other associated chronic liver diseases rather than AIH, such as chronic hepatitis B or C, were excluded. All metabolic liver diseases, such as Wilson’s disease, were also excluded. Patients who had chromosomal disorders or who were treated with medications affecting thyroid function, e.g., amiodarone, lithium, or interferon-alpha, were also excluded.

The collected data included the following:

1. *History taking*: Personal and demographic data: name, age, sex and residency, symptoms suggestive of other autoimmune diseases, manifestations of AIT, and current treatment for AIH. Family history: including parents’ consanguinity, history of similar conditions, or other autoimmune diseases.
2. *Examination*: Vital signs at the time of study enrollment. Anthropometric measurements at the time of study enrollment, including weight, height, and body mass index. They were plotted on Egyptian growth curves [15]. Thorough examination of the thyroid gland. Complete abdominal examination for hepatomegaly, splenomegaly, and ascites. General examination for any stigmata of liver cell failure
3. *Investigations (were performed at the time of study enrollment)*: Basic labs: complete blood count and full liver function tests. Investigations specific for AIH: serum total immunoglobulin G (IgG) and autoantibodies: anti-nuclear antibody (ANA), anti-smooth muscles antibody (ASMA), anti-liver kidney microsomal antibody type 1 (LKM-1), anti-mitochondrial antibody (AMA) and anti-neutrophil cytoplasmic antibody (ANCA). The type of AIH was determined according to the associated positive antibodies at a presentation where ANA and /or ASMA are positive in AIH-1, and anti-LKM-1 is positive in AIH-2. Thyroid workup: Quantitative measurement of FT3 (Diasino®, REF: DS177704, Zhengzhou, China), FT4 (Diasino®, REF: DS177705, Zhengzhou, China), TSH (Diasino®, REF: DS177701, Zhengzhou, China), anti-TPO (EDI™, REF: KT 833, San Diego, USA) and anti-TG (EDI™, REF: KT 832, San Diego, USA) was done using Enzyme Linked Immuno-Sorbent Assay technique (ELISA). A peripheral venous blood sample (5 ml) was collected under complete aseptic conditions from all patients on plane tubes. The serum was separated from the blood after clotting and centrifugation. The serum samples were stored at -20 °C.

Enzyme-Linked Immuno-Sorbent Assay (ELISA) technique principle for FT3 and FT4 detection was the competition principle. At the same time, the sandwich technique was performed for TSH, anti-TPO, and anti-TG detection.

### Thyroid ultrasound

Ultrasound imaging of the thyroid gland was conducted using a Philips Affiniti 70 system with a 12 MHz linear array transducer. Both transverse and longitudinal sweeps were obtained, and Doppler imaging was applied to assess vascularity. Echogenicity was classified as hypoechogenic (decreased brightness relative to adjacent musculature), isoechogenic, or hyperechogenic (increased brightness). Patterns suggestive of autoimmune thyroiditis included a heterogeneous, diffusely hypoechogenic gland with micronodulation. Thyroid scans were done for patients with abnormal thyroid profiles, borderline values, and positive anti-TPO or anti-TG antibodies.

### Hormonal assays

Thyroid testing was performed using ELISA kits according to manufacturer’s protocols (Diasino, Zhengzhou, China). The free T3 assay had a detection range of 1.0-50pmol/L, the free T4 assay 0.25-30ng/dL, the TSH assay 0.005-60μIU/ml, and the anti-TPO and anti-TG assays 10-1000IU/ml.

### Liver function tests

Hepatic biochemical activity was classified based on serum alanine aminotransferase (ALT), aspartate aminotransferase (AST), and gamma glutamyl-transferase (GGT) levels. Complete biochemical remission thresholds were defined as normalizations of both ALT (< 30 U/L for girls and < 40 U/L for boys) as well as IgG levels.

The criteria and tests used to make diagnoses of the additional autoimmune diseases encountered beyond suspected autoimmune thyroiditis.

Relevant conditions and diagnostic standards applied include:

- “Celiac disease: anti-tissue transglutaminase antibodies, in addition to villous atrophy seen grossly by upper GIT endoscopy and histologically by duodenal biopsies.
- Inflammatory bowel diseases: endoscopic visualization plus histologic evidence
- Primary sclerosing cholangitis was diagnosed by magnetic resonance cholangiopancreatography (MRCP)
- Type 1 diabetes mellitus: islet cell autoantibodies, fasting plasma glucose ≥ 126 mg/dL
- Systemic lupus erythematosus: fulfilled ≥ 4 ACR classification criteria
- Skin disorders: cutaneous examination and biopsy as warranted”.
- Diagnosis of FMF had been defined by the presence of at least one major; or at least two minor criteria. Identification of biallelic MEFV pathogenic variants on molecular genetic testing confirmed the diagnosis in some cases.

### Statistical methods

Data were collected and tabulated. Statistical Package for Social Science (SPSS) program version 22 was used for data analysis. Mean and standard deviation (SD) or median and interquartile range (IQR) were estimates of quantitative data as age and laboratory parameters, while frequency and percentage were estimates of qualitative data as sex and associated autoimmune diseases. Differences in biochemical characteristics were tested by students’ paired and unpaired t-test, Mann–Whitney U test, or Wilcoxon test for quantitative data, and by Chi-square test for qualitative data. A two-sided *P* value < 0.05 was considered statistically significant. Differences in AIH remission vs activity status at enrolment between patients with and without autoimmune thyroiditis were tested using the Chi-square test. Remission was classified as either a complete biochemical response or histologic resolution, as defined earlier. Patients not meeting the criteria for remission were categorized under AIH activity. Any difference in the distribution of remission vs activity between the autoimmune thyroiditis positive and negative groups was interpreted as a potential association between thyroiditis and hepatic disease activity.

## Results

This cross-sectional study included 58 children with AIH. Forty patients (69%) were females, with female to male ratio of 2.2:1. Mean ± SD for their age at the time of study enrollment was 11.3 ± 4.5 years, ranging from 1.5 to 18 years.

History of lethargy and poor linear growth were the most common symptoms suggestive of hypothyroidism (43.1%), followed by pubertal delay (41.9%), fatigue (41.4%), hirsutism (31%) and impaired school performance (31%). Headache was the most frequent symptom suggestive of hyperthyroidism (32.8%), followed by polyphagia (27.6%), polyuria (19%), weight loss (17.2%), and palpitations (15.5%). Tremors were present in 3 patients (5.2%). Hyperthermia/ heat intolerance/ thyroid swelling was found in only one patient (1.7%).

Family history of autoimmune diseases in first-degree relatives was reported in 11 patients (19%), including AIH, AIT, rheumatoid arthritis, SLE, type I DM, sclerosing cholangitis, FMF, and alopecia areata.

At the time of study enrollment, all patients had normal heart rates except five patients (8.6%), who had tachycardia, and three patients (5.2%), who had bradycardia. Three patients (5.2%) had tachypnea, and five (8.6%) had a fever. Fourteen patients (24.1%) had systolic blood pressure above the 95th percentile, and 12 patients (20.7%) had diastolic blood pressure above the 95th percentile.

Mean ± SD of body weight at the time of study enrollment was 38.7 ± 18.3 kg ranging from 7.5–83 kg. Mean ± SD of height was 133.3 ± 23.3 ranging from 70–171 cm. Mean ± SD of BMI was 20.4 ± 4.7 kg/m^2^ ranging from 12.6–32.4 kg/m2. Six patients (10.3%) had low body weight with their weight < 3rd percentile. Twelve patients (20.7%) had short stature and two patients (3.4%) had their BMI < 3rd percentile. Only one patient (1.7%) was overweight, and two patients (3.4%) had a BMI > 97th percentile.

Hepatomegaly was detected in 32 patients (55.2%), and five patients (8.6%) had ascites. One patient (1.7%) had goiter. None of the patients had exophthalmos.

Forty patients (69%) had type I AIH (positive ANA and or ASMA), 15 patients (25.9%) had type II AIH (positive anti-LKM-1 antibody), and 3 patients (5.1%) had seronegative AIH.

At the time of enrollment to the study, 28 patients (48.3%) were in activity (out of them, 7 patients were newly diagnosed cases) while 30 patients (51.7%) were in biochemical remission; out of those, 13 patients achieved both biochemical and immunological remission.

**Fig. 1:**
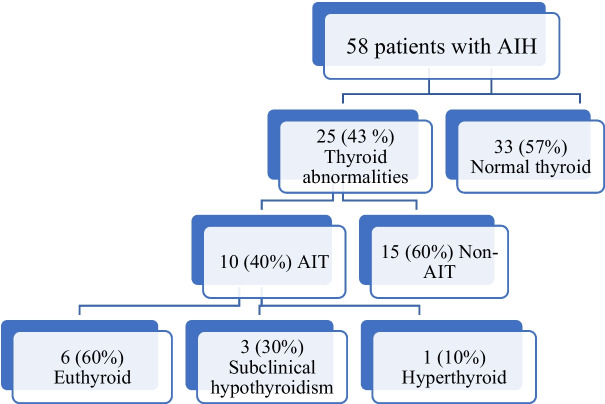
Frequency of patients with thyroid abnormalities, autoimmune thyroiditis and their thyroid status in children with autoimmune hepatitis. AIH: Autoimmune hepatitis, AIT: Autoimmune thyroiditis

To screen for associated AIT, FT3, FT4, TSH, anti-TPO, and anti-TG were done for all patients. Table 1 shows the results of the thyroid profile done for all patients. Figure 1 shows that 25 patients (43%) had thyroid abnormalities in their thyroid profiles, autoantibodies, thyroid ultrasounds, or thyroid isotopic scans. Out of the 25 patients with thyroid abnormalities, ten patients (40%) were diagnosed with AIT.

**Table 1:** Mean ± SD for thyroid profile and autoantibodies done for 58 AIH patients & pubertal status for each enrolled patient

Parameter	Normal range	Result Mean ± SD or Median (IQR)	Min–Max	Number of patients with increased level (%)	Number of patients with decreased level (%)
Free T3	3.3–7.5 pmol/L	4.6 (0.63)	3.1 – 71.7	2 (3.4)	1 (1.7)
Free T4	11.5–23.8 pmol/L	20 ± 7.3	13.3 – 57.4	8 (13.8)	0 (0)
TSH	0.37–5.10 μIU/mL	3.3 ± 1.9	0 – 9.1	9 (15.5)	3 (5.2)
Antithyroid peroxidase	≤ 35 U/mL	13.3 ± 8.4	2.1 – 52.8	1 (1.7)	
Antithyroglobulin	≤ 50 U/mL	25.6 ± 11.9	8.3 – 57.9	2 (3.4)	
**Pubertal Status**					
Prepubertal	7 Patients (12.1%)				
Peripubertal	41 patients (70.7%)				
Postpubertal	10 patients (17.2%)				

*SD* Standard deviation, *T3* Triiodothyronine, *T4* Tetraiodothyronine, *TSH* Thyroid stimulating hormone

Other 15 patients not diagnosed as AIT: 6 of them had subclinical hypothyroidism, one patient with decreased FT3 but with normal FT4 and TSH, one patient with low TSH, five patients with increased FT4 with normal TSH and normal FT3 except for one patient only had also increased FT3, one patient with normal thyroid gland US but with abnormal thyroid scan showing mild diffuse goiter with normal uptake with normal thyroid profile and negative autoantibodies. Another patient had a mildly enlarged gland in the US, and a thyroid scan showed a mildly enlarged gland with fine nodular goiter with normal thyroid uptake.

Thyroid status of AIT patients showed that six patients (60%) were euthyroid, three patients (30%) had subclinical hypothyroidism, and only one patient (10%) was hyperthyroid. Table 2 describes children with associated AIT’s clinical, autoimmune profile, and AIH disease activity. Out of the 10 with AIT, only one patient had Graves disease (GD), and the other nine patients had HT.

**Table 2:** Age, sex, AIH status, thyroid status and thyroid radiological findings of AIT patients

Pt	Age (ys)	Sex	AIH status	Free T3	Free T4	TSH	Thyroid status	Anti-TPO	Anti-TG	Thyroid US	Thyroid scan
1^a^	10	F	Activity	N	N	↑	Subclinical hypo-thyroidism	-ve	-ve	N	Chronic thyroiditis
2	13	F	Activity	N	N	↑	Subclinical hypo-thyroidism	-ve	-ve	N	Hyper-functioning gland
3	7	M	Biochemical remission	↑	N	↓	Hyper-thyroid	-ve	-ve	N	Hyper-functioning gland
4	9	F	Biochemical remission	N	↑	N	Euthyroid	-ve	-ve	N	Hyper-functioning gland
5^a^	17	F	Biochemical remission	N	N	N	Euthyroid	+ ve	-ve	N	Not done due to parental refusal
6	15.5	F	Biochemical remission	N	N	N	Euthyroid	-ve	-ve	N	Hyper-functioning gland
7	18	M	Activity	N	N	↑	Subclinical hypo-thyroidism	-ve	+ ve	Mild diffuse goiter	Mild diffuse goiter with hyper-functioning gland
8	12	F	Activity	N	↑	N	Euthyroid	-ve	-ve	N	Relatively low homogenous thyroid uptake
9	12	F	Activity	N	↑	N	Euthyroid	-ve	+ ve	N	Hyper-functioning gland
10	11	F	Activity	N	N	N	Euthyroid	-ve	-ve	Mild diffuse goiter	Mild diffuse goiter with normal thyroid uptake

↑: Increased above normal level

↓: Decreased below normal level

-ve: Negative

+ ve: Positive

*AIH* Autoimmune hepatitis, *AIT* Autoimmune thyroiditis, *Anti-TG* Antithyroglobulin, *Anti-TPO* Antithyroid peroxidase, *F* Female, *M* Male, *N* Normal, *Pt* Patient, *T3* Triiodothyronine, *T4* Tetraiodothyronine, *TSH* Thyroid stimulating hormone, *US* Ultrasound

^a^Patient 1 with Hashimoto’s thyroiditis has a paternal aunt with hypothyroidism requiring levothyroxine treatment. Meanwhile Patient 5 reports a brother with Graves’ disease

Patients with and without AIT were compared regarding demographic, clinical, and laboratory data (Table 3). Of all symptoms and signs suggestive of thyroid dysfunction, the following manifestations were the only statistically present in patients with AIT: goiter (*p*-value = 0.027), impaired school performance (*p*-value = 0.03), hyperthermia (*p*-value = 0.027) and heat intolerance (*p*-value = 0.027).

**Table 3:** Comparison between AIH patients with and without associated AIT

Parameters	Patients without AIT *N* = 48	Patients with AIT *N* = 10	Total *P*-value	Detailed *P*-value
Sex: N (%)				
Females	33 (68.7)	8 (80)	0.48	
Males	15 (31.3)	2 (20)		
Age in years; mean ± SD	11 ± 4.7	12.5 ± 3.5	0.37	
Age of onset in years; mean ± SD	6.6 ± 4.1	8.3 ± 3.3	0.22	
Mode of presentation: N (%)				
Acute hepatitis	24 (50)	7 (70)	0.69	0.23
Insidious onset	7 (14.6)	1 (10)		0.69
Acute liver cell failure	10 (20.8)	1 (10)		0.42
Complications of chronic liver disease	7 (14.6)	1 (10)		0.69
Symptoms & signs of thyroid affection				
Goiter	0 (0)	1 (10)	0.027^*^	
Impaired school performance	12 (25)	6 (60)	0.03^*^	
Hyperthermia	0 (0)	1 (10)	0.027^*^	
Heat intolerance	0 (0)	1 (10)	0.027^*^	
Positive family history of AIH	3 (6.3)	1 (10)	0.54	
Positive family history of AIT	2 (4.2)	1 (10)	0.44	
Family history of ADs	8 (16.7)	3 (30)	0.32	
Autoimmune disease association	18 (37.5)	3 (30)	0.65	
Weight in Kg < 3^rd^ percentile	6 (12.5)	0 (0)	0.23	
Weight > 97^th^ percentile	1 (2.1)	0 (0)	0.62	
Height in cm < 3^rd^ percentile	10 (20.8)	2 (20)	0.92	
Height > 97^th^ percentile	1 (2.1)	0 (0)	0.62	
BMI kg/m^2^ < 3^rd^ percentile	2 (4.2)	0 (0)	0.48	
BMI > 97^th^ percentile	1 (2.1)	1 (10)	0.23	
Total Ig G at presentation	2755.5 ± 1208	3200.1 ± 440.7	0.41	
Total Ig G at study enrollment	1819.7 ± 1208.7	1866 ± 805.7	0.66	
Positive ANA at study enrollment	6 (12.5)	0 (0)	0.58	
Positive ASMA at study enrollment	23 (47.9)	6 (60)	0.49	
Positive anti-LKM antibody at study enrollment	7 (14.6)	0 (0)	0.19	
Positive AMA at study enrollment	0 (0)	1 (10)	0.027^*^	
Positive ANCA at study enrollment	4 (8.3)	1 (10)	1	
Type of AIH:				
Seronegative	2 (4.2)	1 (10)	0.35	0.42
Type I	32 (66.7)	8 (80)		0.42
Type II	14 (29.2)	1 (10)		0.19
Remission at the time of the study	26 (54.2)	4 (40)	0.42	

Chi Square, Mann Whitney and independent sample t tests are used

*ADs* Autoimmune diseases, *AIH* Autoimmune hepatitis, *AIT* Autoimmune thyroiditis, *AMA* anti-mitochondrial antibodies, *ANA* anti-nuclear antibody, *ANCA* anti-neutrophil cytoplasmic antibodies, *Anti-LKM-1* anti-liver kidney microsomal type 1 antibody, *ASMA* anti-smooth muscle antibody, *BMI* Body mass index, *IgG* immunoglobulin G, *SD* Standard deviation, *T3* Triiodothyronine, *T4* Tetraiodothyronine, *TG* Thyroglobulin, *TPO* thyroid peroxidase, *TSH* Thyroid stimulating hormone

*P* value is significant at level < 0.05

“*” Significant

AMA was significantly more prevalent in patients with AIT (*P* = 0.027) (Table 3). There was no statistically significant difference between the two groups regarding AIH activity, raising the importance of screening for associated AIT for all patients with AIH, even if they are in remission.

Finally, we found that out of 58 patients of AIH, 28 patients (48.3%) had other comorbid autoimmune disorders. Seven patients had two associated autoimmune disorders, and one patient had three associated autoimmune disorders, thus raising the total number to 37 associated autoimmune disorders in 28 patients, as shown in Table 4. The most common autoimmune disease in children with AIH was AIT (17.2%).

**Table 4:** Frequency of other autoimmune diseases in children with AIH (*n* = 58)

Associated autoimmune diseases	N (%)
**Hepatobiliary & gastrointestinal tract**	
Primary sclerosing cholangitis	7 (12.1)
Ulcerative colitis	2 (3.4)
Crohnʼs disease	1 (1.7)
Celiac disease	1 (1.7)
**Endocrinal**	
Autoimmune thyroiditis	10 (17.2)
Type I DM	1 (1.7)
**Rheumatological**	
SLE	5 (8.6)
FMF	1 (1.7)
**Skin**	
Alopecia	6 (10.3)
Vitiligo	1 (1.7)
Psoriasis	1 (1.7)
Discoid lupus erythematosus	1 (1.7)

*AIH* Autoimmune hepatitis, *DM* Diabetes mellitus, *FMF* Familial medeterinian fever, *SLE* systemic lupus erythematosus

## Discussion

Like other autoimmune diseases, AIH can be associated with one or more autoimmune disorders, either organ-specific or non-organ-specific. Different studies estimated the frequency of the association of AIH and AIT in adult patients around 10% [16]. Our study aimed to detect the frequency of AIT among Egyptian children with AIH.

History of autoimmune disease in the patient himself or in any family members can be a clue in the diagnosis of AIH. In our study, a family history of autoimmune diseases was reported in 11 patients (19%) of our study group. Concomitant autoimmune diseases were reported in 28 patients (48.3%). Khoury et al. reported concomitant autoimmune diseases in 20 and 50% of adult and pediatric AIH patients, respectively [3]. Meanwhile, other authors reported associated autoimmune disease in 23.5% of patients in their study [17].

In our study, AIT was the most commonly associated extrahepatic autoimmune disease with AIH, as it was detected in 10 children (17.2%). Similarly, several studies reported that AIT was the most familiar extrahepatic autoimmune disease associated with AIH (including HT, GD, and unspecified autoimmune thyroiditis) [6, 9, 18–20]. This demonstrates the importance of screening AIH patients for early detection of associated AIT.

Growth failure is a common problem and multifactorial in many children with chronic diseases, including children with chronic liver disease. Twelve patients (20.7%) in the current study had short stature with height < 3^rd^ percentile for age; 2 were diagnosed as AIT. Previous researchers reported that critical contributory factors to growth failure in these patients include increased energy needs, energy loss, malabsorption, decreased energy intake, anorexia, pain, vomiting, and inflammatory cytokines. Oral corticosteroid therapy is known to be associated with a delay in growth and puberty in children [21, 22].

In the present study, AIT was more common in type I AIH than type II, although the difference was not statistically significant. However, A previous report demonstrated that AIT was more common in type 2 AIH [23]. Potential Explanations for Increased AIT in Type 1 AIH could be due to differing genetic risk alleles and additional non-HLA polymorphisms that may promote varied susceptibility for thyroid targeting between AIH subtypes in addition to unique autoantibody profiles and epitope spreading [24]. Also, age of onset and pubertal influences likely contribute, as type 2 AIH manifests earlier when thyroiditis relies on hormonal changes in addition to the early immunosuppressant exposure after type 2 diagnosis may protectively dampen mechanisms underlying AIT [25].

Sixty percent of our patients with AIT were euthyroid, while 30% had subclinical hypothyroidism, and only 10% had hyperthyroidism; this explains the importance of screening for AIT in AIH patients even if there are no symptoms suggestive of thyroid affection as most patients were euthyroid. Similarly, Wasniewska and co-authors discovered that 52.1% were euthyroid, 19.2% had subclinical hypothyroidism, 22.2% had overt hypothyroidism, and only 6.5% had hyperthyroidism [26]. This high percentage of euthyroid AIT in AIH patients emphasizes the importance of AIT screening in AIH patients.

Some previous investigators reported different results, as 21% were euthyroid, 42% had subclinical hypothyroidism, and 37% had overt hypothyroidism [27].

Out of our ten patients with AIT, only one patient (10%) had GD and the other nine (90%) had HT. This was in accordance with Aversa et al. who reported that GD is much less common than HT, especially in pediatric age [28]. Moreover, Salerno et al. stated that HT is the most common form of thyroiditis in pediatric age [29].

In most chronic illnesses, defects arise in thyroid hormone metabolism, resulting in sick euthyroid syndrome or non-thyroidal illness. This is characterized by a normal total T4, normal/high FT4, low total T3, low FT3, and an elevated reversed T3 (rT3) [30]. This mechanism can explain the abnormal thyroid profile found in 15 of our patients and not proved to have AIT yet. Similarly, Huang and Liaw reported that liver diseases are frequently associated with thyroid test abnormalities or dysfunctions, particularly elevation of thyroxine-binding globulin and thyroxine [31]. Neglect of these facts may result in misdiagnosis of associated thyroid diseases and thereby cause errors in patient care.

We found that there was a statistically significant difference between patients with and without hyperthyroidism regarding goiter (*p* value = 0.27), impaired school performance (*p*-value = 0.03), hyperthermia (*p*-value = 0.27), and heat intolerance (*p*-value = 0.27) but all other symptoms and signs of thyroid affection showed no statistical significant patients meaning that screening for all AIH patients is essential even if they are asymptomatic.

The main limitation of our study is the relatively small number of included patients and the cross-sectional nature of the study design. Furthermore, thyroid scans and ultrasounds were only performed on patients with abnormal or borderline thyroid profiles. Despite these limitations, this study represents one of the largest studies performed on pediatric patients with AIH in Egypt.

## Conclusion

Autoimmune hepatitis in Egyptian children is commonly associated with other autoimmune diseases. Autoimmune thyroiditis is the most common condition associated with AIH in pediatric patients. As it is not usually clinically manifesting, regular screening for AIT in children with AIH is mandatory.

## Data Availability

The datasets used and/or analyzed during the current study are available from the corresponding author on reasonable request.
